# Enhanced Apoptotic Effects in MDA-MB-231 Triple-Negative Breast Cancer Cells Through a Synergistic Action of Luteolin and Paclitaxel

**DOI:** 10.7759/cureus.65159

**Published:** 2024-07-22

**Authors:** Shabnam Tamanna, Elumalai Perumal, Jeevitha Rajanathadurai

**Affiliations:** 1 Department of Pharmacology, Saveetha Dental College and Hospitals, Saveetha Institute of Medical and Technical Sciences, Chennai, IND; 2 Cancer Genomics Laboratory, Centre for Global Health Research, Saveetha Medical College and Hospitals, Saveetha Institute of Medical and Technical Sciences, Chennai, IND

**Keywords:** apoptosis, bcl-2, luteolin, paclitaxol, breast cancer

## Abstract

Background and aim: According to reports on cancer incidence in 2020, breast cancer became the leading malignancy among women worldwide. This multistep disease involves genetic and environmental factors. Paclitaxel, a naturally occurring antimitotic substance, is a widely used chemotherapeutic drug for treating various human malignancies, including breast cancer. However, its major drawback is its extensive toxicity. This limitation can be mitigated through combination therapy with natural products like luteolin. Studies suggest that luteolin has anticancer properties, as it inhibits cancer cell growth and induces apoptosis in breast, lung, and colon cancers. This study aims to investigate the synergistic anticancer effects of combining luteolin and paclitaxel on breast cancer cells.

Methods: Breast cancer cell line (MDA-MB-231) was utilized for this study. 3-(4,5-Dimethylthiazol-2-yl)-2,5-diphenyltetrazolium bromide (MTT) assay was then conducted to check the cell viability. This was followed by a morphology study conducted under a phase contrast microscope. Morphological analysis revealed pronounced cell shrinkage and membrane blebbing, indicative of apoptosis when treated with the combination at their IC50 values. Gene expression results further confirmed the anticancer properties by showing significant downregulation of the B-cell lymphoma-2 (BCL-2) anti-apoptotic gene. These findings suggest that the luteolin-paclitaxel combination exerts a synergistic effect, enhancing anticancer activity in breast cancer cells. Reverse transcriptase polymerase chain reaction (RT-PCR) was done to analyze the genes involved in apoptosis. Finally, the data collected was statistically analyzed to confirm the reliability of the study.

Results: The combination of 1 μM/ml of paclitaxel and increasing concentrations of luteolin showed a great percentage of reduction in cell viability and the IC50 value of luteolin concentration was around 40 μM/ml. The morphology study revealed that the cancer cells showed shrinkage and blebbing on treatment with 40 μM/ml. At the same IC50 concentration, the combination of luteolin and paclitaxel resulted in a significant downregulation of BCL-2 mRNA expression in breast cancer cells compared to luteolin alone.

Conclusion: The combination of paclitaxel and luteolin has a synergistic effect on breast cancer cells and shows potential as a treatment for various cancers. Given these promising results, the paclitaxel and luteolin combination could be developed into a potent therapeutic strategy for treating various cancers. Future research should include in vivo studies to further assess the therapeutic potential and safety profile of this combination.

## Introduction

Breast cancer is a serious health issue worldwide that affects millions of people each year and provides a challenging environment for scientific research. As per the data collected by the World Health Organization (WHO) in 2020, all over the world, around 700,000 individuals lost their lives due to breast cancer, with around 2.5 million women receiving a diagnosis [1-3]. Due to its extensive prevalence and mortality rate, treatment of breast cancer is very crucial [4]. The currently employed treatment modalities have shown excellent efficiency in reducing the number of cancer-related deaths.

Carboplatin, taxanes, anthracyclines, and many other drugs are currently being utilized in conjunction with chemotherapy. Among chemotherapeutic agents, paclitaxel, due to its wide application in breast cancer, is the drug chosen for this study. Although it is being studied for the treatment of numerous tumors, paclitaxel is currently approved for use in the treatment of lung cancer, ovarian cancer, and Kaposi's sarcoma. Although all of these methods of treatment are highly effective, they can extensively deteriorate the quality of life of the individual. Due to such adverse effects, it is now crucial to find safer alternatives. Natural resources have been used in traditional medicine for ages [5-7]. Luteolin is one such plant-derived, thermostable flavonoid. Traditional Chinese medicine has long embraced the use of luteolin-rich plants for treating conditions like hypertension, rheumatoid arthritis, and cancer. Luteolin has various biological effects, including anti-inflammatory, anti-allergic, and anticancer properties [8]. 

The anticancer activity of luteolin is linked to causing apoptosis, which includes regulating redox, causing DNA damage, and affecting protein kinases to stop cancer cell growth and prevent metastasis and angiogenesis. It also suppresses the cell growth cycle and promotes the cellular apoptotic cycle [9]. The synergistic anticancer effect of paclitaxel and luteolin or other flavonoids is being researched on a wide scale to find a combination that would reduce the harm caused by paclitaxel on normal cells and at the same time also enhance tumor suppression. Earlier in vitro research showed that co-administration of luteolin and paclitaxel in MDA-MB-231 breast cancer cells induces apoptosis through the suppression of signal transducer and activator of transcription 3 (STAT3) gene [10]. This study aims to investigate the synergistic apoptotic effects of luteolin and paclitaxel on MDA-MB-231 triple-negative breast cancer (TNBC) cells. By combining these agents, we hypothesize that their interaction will result in enhanced cytotoxicity and apoptosis compared to their individual use. Understanding the mechanisms underlying this synergistic action could pave the way for more effective combination therapies, potentially improving outcomes for patients with TNBC. 

## Materials and methods

Maintenance of the cell line

The National Centre Cell Sciences (NCCS), Pune, provided a cell line of breast cancer (MDA-MB-231). The cells were grown in T25 culture flasks that included 10% fetal bovine serum (FBS) and 1% antibiotics in addition to Dulbecco's modified Eagle medium (DMEM). Cells maintained at 37°C in moist surroundings in 5% CO₂. Trypsinization of the cells was done and they were passed after they had reached confluence.

Cell viability assay

The cancerous cell viability after treatment with luteolin and paclitaxel was evaluated using the 3-(4,5-dimethylthiazol-2-yl)-2,5-diphenyltetrazolium bromide (MTT) test. The procedure follows the principle by which cells with metabolic activity change soluble yellow tetrazolium salt into insoluble purple formazan crystals. The 96-well plates containing 5x10³ MDA-MB-231 cells/well were used for the plating. After plating, cells were starved by culturing them in serum-free medium for 3 hours at 37°C. Two flushes with 100μl of medium without serum were then performed 24 hours later. Post starvation, cells were exposed to varying doses of luteolin and paclitaxel over 24 hours. After the treatment was completed, 100μl of the media from the treated and untreated cells were added to each well, along with DMEM (0.5 mg/ml) containing MTT. Then, the cells were maintained at 37°C for 4 hours in the CO₂ incubator. Subsequently, the MTT-containing medium was eliminated, and the cells were cleaned using a single phosphate-buffered saline (PBS) wash. After dissolving the formed formazan crystals in 100μl of dimethyl sulfoxide (DMSO), the mixture was allowed to settle in the dark for one hour. A micro enzyme-linked immunosorbent assay (ELISA) plate reader was then used to measure the color intensity at 570 nm. The proportion of control cells grown in a medium without serum serves as an estimate for the total number of living cells. Now 100% of the cells were viable in the control media when no treatment was applied. The following formula is used to determine the cell viability: 

% cell viability = A570 nm of treated cells/A570 nm of control cells×100

Morphology analysis

Based on the MTT assay, we chose the best doses (IC50: luteolin, 40 µM/ml; paclitaxel, 1 µM/ml) for further research. A phase contrast microscope is used to examine changes in cell morphology. Six well plates containing 2×10^5^ cells were then treated for 24 hours with paclitaxel and luteolin for the treatment of cancer cells. The media was taken out of the cells after the incubation period and they were given one wash in PBS at a pH of 7.4. With the use of an inverted phase contrast microscope, the plates were examined. 

Real-time polymerase chain reaction (PCR)

Using real-time PCR, the B-cell lymphoma-2 (BCL-2) gene expression was examined. Using Trizol Reagent (Sigma-Aldrich, Burlington, MA), the total RNA was extracted according to the established technique. PrimeScript, 1st strand cDNA synthesis kit (Takara, Shiga, Japan) was employed to reverse-transcribe 2 μg of RNA to create cDNA. Particular primers were used to amplify the targeted genes. By using the GoTaq® qPCR Master Mix (Promega, Madison, WI), which contains SYBR green dye along with other PCR components, the PCR process was done. RT-PCR was performed in a CFX96 PCR system (Biorad, Hercules, CA, USA). The data were analyzed using the 2−∆∆CT method to generate the fold change graph.

 Statistical analysis

The entire data were examined using SPSS Statistics, version 26 (IBM Corp., Armonk, NY), first with one-way analysis of variance (ANOVA) and then Student's t-test. The findings are displayed in triplicate as mean ± SD. A value of p<0.05 was selected for statistical significance.

## Results

Cytotoxic effects of paclitaxel on breast cancer cells

**Figure 1:**
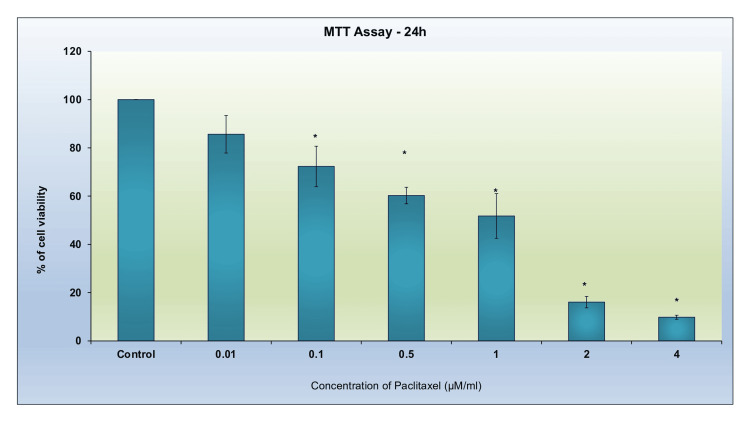
Cytotoxic impacts of paclitaxel on breast cancer cells (MDA-MB-231). The MTT test was employed to evaluate the viability of the cancer cells after a 24-hour treatment with paclitaxel (0.01-4 μM/ml). The three independent experiments' mean ± SD, each carried out in triplicate, are shown in the data. Significant differences (p < 0.05) are indicated by asterisks (*) as compared to the untreated control group. MTT: 3-(4,5-dimethylthiazol-2-yl)-2,5-diphenyltetrazolium bromide.

The MTT assay was conducted with increasing concentrations of paclitaxel (0.01-4μM/ml) and it is evident from the results in Figure 1 that with the increase in the concentration of paclitaxel, the percentage of viable cells decreased. The results were compared with the control (untreated) group. The inhibition capacity (IC50) value, which is the concentration that renders 50% of the cells non-viable, was found to be 1μM/ml (Figure 1). This concentration provided a measure of the potency of the compound; so it was further used to check the combined effect of paclitaxel and luteolin on the cancer cell line. 

 

Cytotoxic effects of luteolin and paclitaxel combination in breast cancer cells

**Figure 2:**
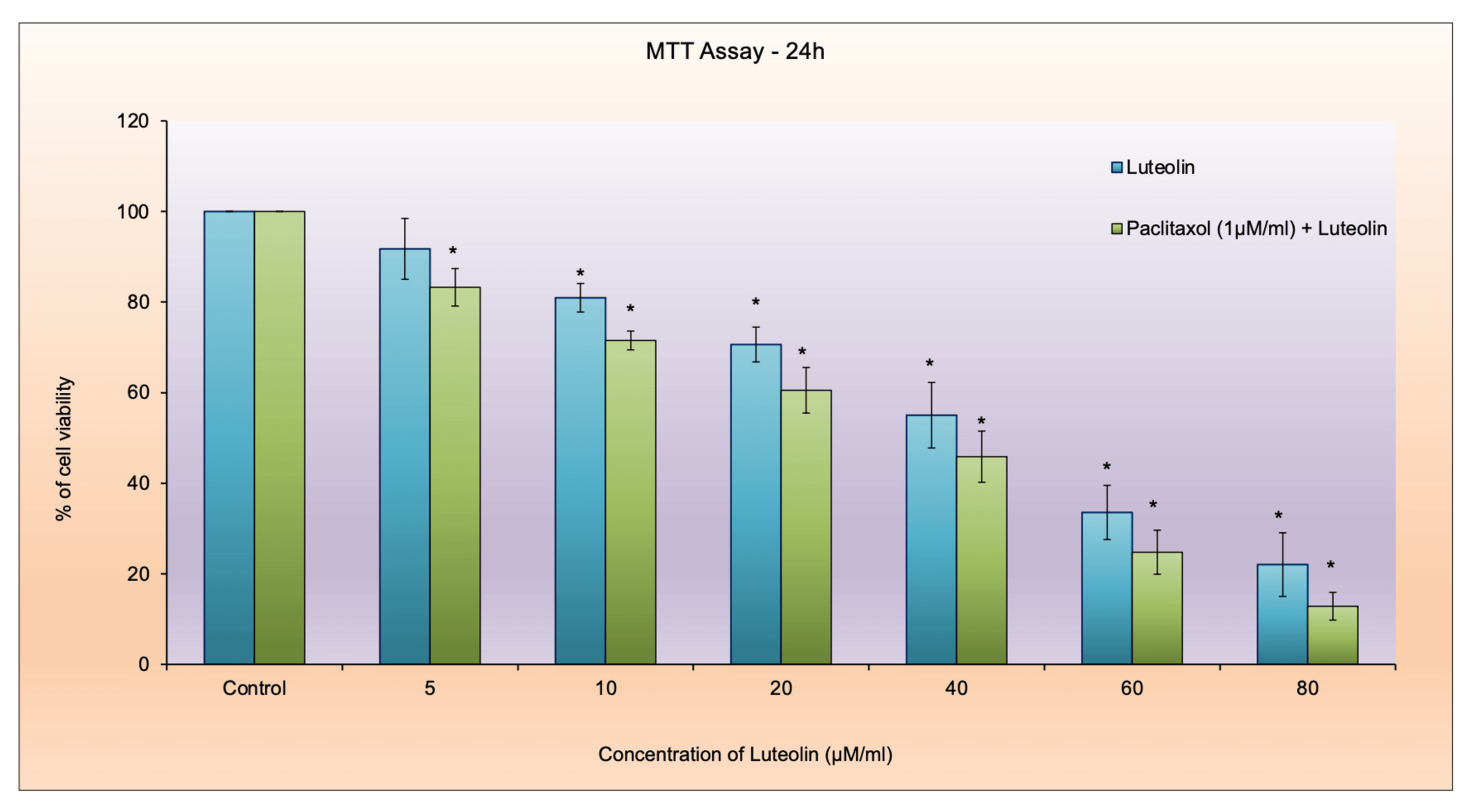
Cytotoxic effects of luteolin and paclitaxel on breast cancer cells (MDA-MB-231) Cell viability was assessed using the MTT assay after treating the cells with luteolin and paclitaxel extract (5-80 μM/ml) for 24 hours. The data are expressed as mean ± SD from three independent experiments, each conducted in triplicate. Asterisks (*) indicate significant differences compared to the control untreated group (p < 0.05). MTT: 3-(4,5-dimethylthiazol-2-yl)-2,5-diphenyltetrazolium bromide.

The MTT experiment was conducted using increasing concentrations of luteolin (5-80 μM/ml) in combination with a fixed concentration of 1 μM/ml paclitaxel to evaluate their cytotoxic effects on cancer cells. As depicted in Figure 2, the data clearly demonstrate that as the concentration of luteolin increased, the percentage of cell viability decreased correspondingly. This indicates that luteolin exhibits a dose-dependent cytotoxic effect on the cancer cells. From the data analysis, the inhibitory concentration 50 (IC50) value for luteolin was determined to be 40 μM/ml, meaning that at this concentration, luteolin reduced cell viability by 50%. This finding highlights the potency of luteolin as an anticancer agent. Further analysis revealed that the cytotoxic effects of the combination treatment (luteolin and paclitaxel) were comparable to those of standard paclitaxel treatment alone, with only a marginal difference of approximately 10%. This suggests that combining luteolin with paclitaxel does not significantly diminish the efficacy of paclitaxel; instead, it maintains a similar level of cytotoxicity and may potentially offer additional therapeutic benefits, such as reduced side effects or overcoming drug resistance.

Morphology analysis

**Figure 3:**
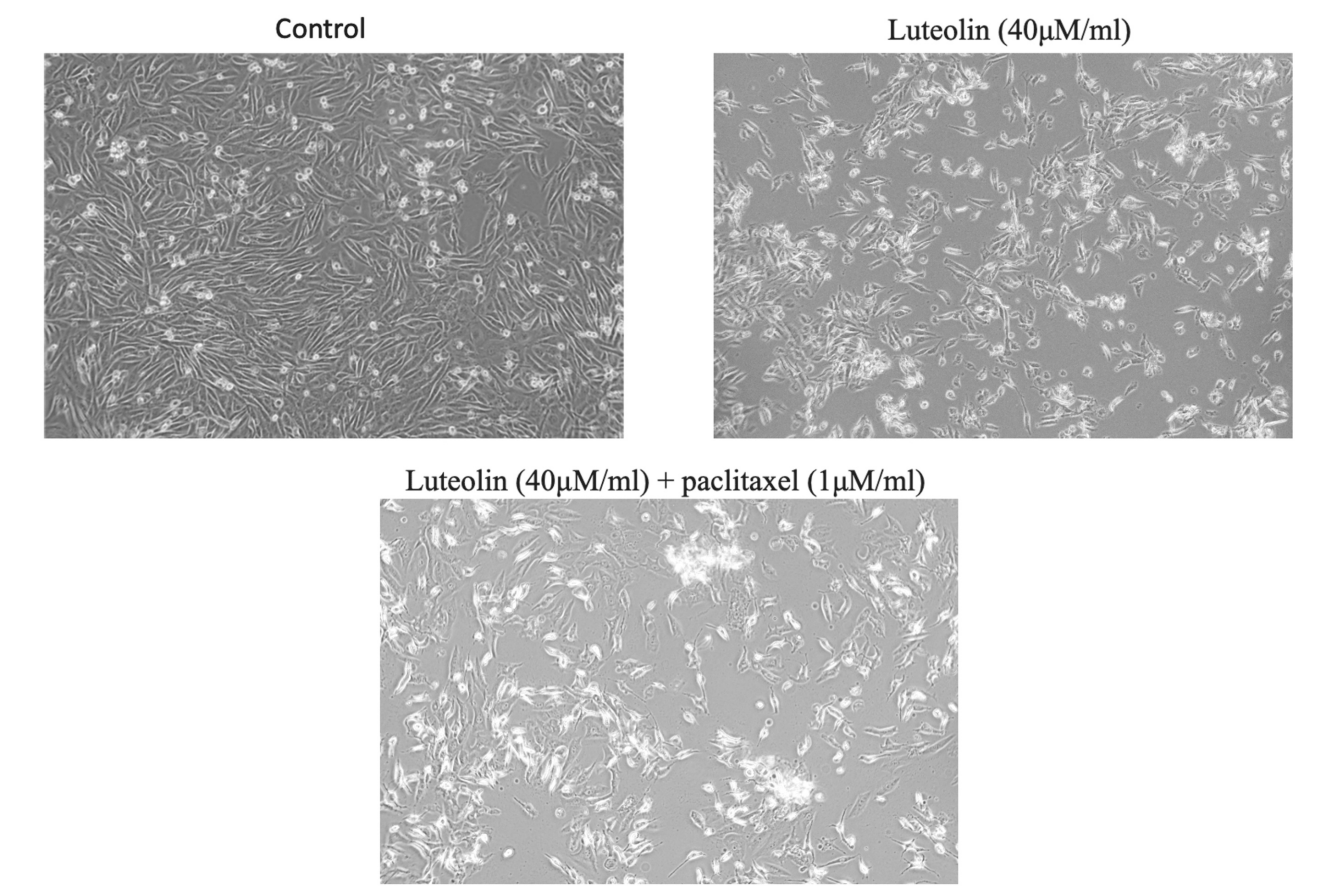
Effect of luteolin and paclitaxel on cell morphological changes in breast cancer cells. The morphological changes in human breast cancer cells (MDA-MB-231) induced by luteolin and paclitaxel were observed using an inverted phase contrast microscope. Following treatment, there was a noticeable decrease in cell number, and the cells exhibited shrinkage compared to the control group.

Luteolin and paclitaxel (40μM/ml) were applied to cells for 24 hours, after which they were examined using an inverted phase contrast microscope. Upon treatment with 33μM/ml luteolin, fewer cells were present, and those that were present showed signs of cellular shrinking and cytoplasmic membrane blebbing. As visualized in Figure *3*, on further treatment with luteolin (40μM/ml) and paclitaxel (1μM/ml), the cancer cells were scarce with higher levels of apoptosis denoted by the white areas. When compared with the control, the cell line has shown a regressive pattern in their number and growth denoting that luteolin and paclitaxel at their IC50 values show immense anticancer properties (Figure 3). 

Luteolin and paclitaxel inhibit the BCL-2 gene expression in breast cancer cells

**Figure 4:**
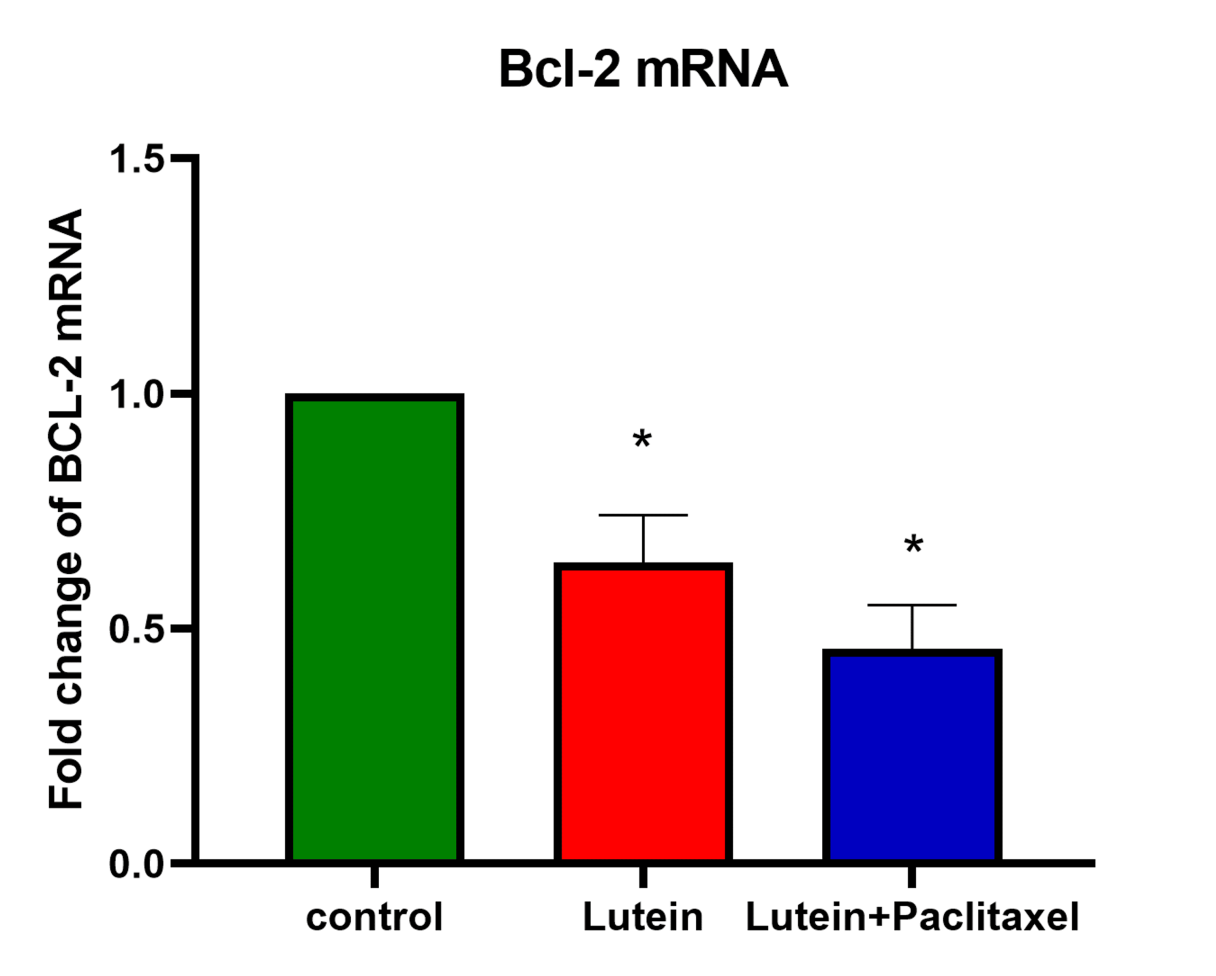
Effect of luteolin and paclitaxel (40μM/ml) on BCL-2 gene expression in breast cancer cell line. The results are expressed as a fold change relative to the control, with the expression of the target gene normalized to glyceraldehyde-3-phosphate dehydrogenase (GAPDH) mRNA expression. Data are expressed as mean ± SD from three independent experiments, each performed in triplicate and represented by a bar. Asterisk (*) represents the statistical significance between untreated and drug-treated groups at p<0.05.

RT-PCR was performed to evaluate the expression levels of the BCL-2 gene in the MDA-MB-231 cell line following treatment with a combination of luteolin and paclitaxel. The BCL-2 gene is known for its anti-apoptotic properties, and its expression levels can provide insight into the apoptotic response of cancer cells to treatment. The study compared the results from cells treated with the drug combination to those from untreated control cells and cells treated with luteolin alone. The data, depicted in Figure 4, revealed that the combination of luteolin and paclitaxel led to a significant downregulation of BCL-2 mRNA expression in the MDA-MB-231 cell line. This suggests a strong induction of apoptosis due to the combined treatment, as the suppression of BCL-2 can promote cell death. In comparison, cells treated with luteolin alone showed a 50% reduction in BCL-2 mRNA expression relative to the control, indicating that luteolin also contributes to the induction of apoptosis but to a lesser extent than the combination therapy. This combination therapy appears to be more effective at promoting apoptosis in MDA-MB-231 cells than luteolin alone, highlighting its potential as a powerful therapeutic strategy against triple-negative breast cancer. 

## Discussion

The majority of the chemotherapy treatments that are often employed are linked to extremely nonspecific cytotoxicity, limited therapeutic indices, and severe side effects. Given its strong antitumor activity against breast cancer, paclitaxel is recommended for the treatment of cancer. However, for cancer patients, paclitaxel chemotherapy's effectiveness is restricted due to neurotoxicity [11]. Hence, it is important to develop cancer chemotherapy approaches that have reduced toxicity and increased tumor response [12]. This study concentrated on assessing the synergistic anti-cancer properties of luteolin and paclitaxel against breast cancer cell line MDA-MB-231. Zhao et al. state that luteolin was presented as a viable treatment option for oesophageal cancer, which was resistant to paclitaxel [13]. Another study that used adult tongue squamous carcinoma cells (SCC-4) found that the combination of luteolin and paclitaxel increased the cytotoxicity of the medication. Regular consumption of the aforementioned flavonoid may therefore reduce the formation of tumors in animal models [14].

The MTT assay results showed that the combination of paclitaxel and luteolin was around 10% more efficient in reducing cell viability of the cancer cells. A similar result that after 24 and 48 hours, luteolin dramatically enhanced cytotoxicity when compared to paclitaxel therapy alone was reported in a previous study [10]. Another prior study using breast cancer cells that were independent of estrogen has demonstrated that coadministration of luteolin with the chemotherapeutic drug paclitaxel increases cytotoxicity [15]. The morphology study showed that after treatment of the cancer cell line with a combination of paclitaxel and luteolin, the cells shrunk in size and expressed cytoplasmic blebbing. In a study where MDA-MB-231 was implanted into nude mice, similar results were observed where a 96.5% decrease in the tumor was observed [10]. The phase contrast morphology results of another study showed that luteolin downregulated epithelial-mesenchymal transformation in paclitaxel-resistant ovarian cancer cells as evident by a reduced mesenchymal phenotype in comparison to untreated cells [16]. 

In complex organisms, apoptosis is crucial for preserving growth and equilibrium because it gets rid of unnecessary or extra cells. Apoptosis, which is frequently dysregulated in malignancies, could be the main way that anti-tumor drugs work. Furthermore, a crucial focus for cancer treatment is the stimulation of apoptosis. The two main routes that induce apoptosis are the intrinsic and extrinsic pathways. This study deals with the intrinsic pathway and the BCL-2 gene expression from the BCL-2 family to observe the anti-apoptotic effect of paclitaxel and luteolin. Structurally similar molecules known as Bcl₂ family proteins can either favorably or negatively influence apoptosis. One important factor in determining cellular homeostasis is the comparative balance of different pro-apoptotic (Bcl-2-associated X protein (BAX), Bcl-2-associated death promoter (BAD)) and anti-apoptotic (BCL-2, BCL-xL, MCL-1) BCL-2 family members [17]. Cancer frequently exhibits dysregulation of the BCL-2 family proteins, including mutations or abnormalities in pro-apoptotic members and overexpression of anti-apoptotic members. In comparison with the control, the PCR results of the study showed a significant downregulation of the BCL-2 gene in the cancerous cell line when treated with paclitaxel and luteolin. In a similar study, the PCR results showed that luteolin- and paclitaxel-treated MDA-MB-231 did not show any significant change in the expression of the BCL-2 gene [10]. By preventing Bax and Bak from oligomerization, the anti-apoptotic BCL-2 gene prevents the mitochondrial apoptotic machinery from being engaged. Apoptotic signaling pathways that malfunction play an important part in the development and distribution of cancer [18]. 

Limitations

Although this study provided substantial data in support of luteolin’s anticancer properties along with paclitaxel, there are certain limitations of the study. As it is a novel study, there is very little data available for comparison. This is a preliminary study and although it shows excellent results, in vivo and clinical studies must be done before it can be released in the market. Despite the small sample size, the study has provided significant data. The study can be performed on a larger scale for more conclusive results.

## Conclusions

The study demonstrated that luteolin and paclitaxel have a synergistic anticancer effect on breast cancer cells by reducing cell viability, inducing significant morphological changes, and downregulating the BCL-2 anti-apoptotic gene. This combination therapy shows promise for further in vivo research and potential development into a plant-based treatment for breast cancer. Future studies should explore other flavonoids and alkaloids for enhanced anticancer activities and test the model on various cancer cell lines to broaden its applicability.
